# Circuitry correlates of negative urgency and suicidality in schizophrenia spectrum disorders: a LASSO regression study

**DOI:** 10.3389/fpsyt.2025.1678555

**Published:** 2025-12-09

**Authors:** Tyler Pia, Enna Sanghvi, Mark Shuquan Chen, Thomas Kim, Matthew J. Hoptman, Anthony O. Ahmed

**Affiliations:** 1 Department of Psychiatry, Weill Cornell Medicine, New York, NY, United States; 2 Department of Psychology, Yale University, New Haven, CT, United States; 3 Division of Clinical Research, The Nathan S. Kline Institute for Psychiatric Research, Orangeburg, NY, United States; 4 Department of Psychiatry, NYU Grossman School of Medicine, New York, NY, United States

**Keywords:** urgency, psychosis, schizophrenia spectrum disorders, suicidality, suicidal ideation, suicidal behavior, LASSO

## Abstract

**Objective:**

Suicidality is highly prevalent with vast public health implications. The association between schizophrenia spectrum disorders (SSDs) and suicidal ideation and related behaviors (SIBs) is well established. One construct that has received less attention is *emotion-based impulsivity* or *urgency*, defined as impulsive action in the context of high positive or negative emotion states. The current study leverages a machine learning approach to examine the association of SSD symptoms, urgency, and suicide risk.

**Method:**

We used least absolute shrinkage and selection operator (LASSO) logistic regression to generate classifications of individuals recruited as high-risk (n=14) and low-risk (n=16) for SIBs (n=30, M_age_=41.07, SD_age_=10.95, 13.3% female) Model 1 included demographics, childhood trauma, SSD symptoms, global cognitive ability, and urgency. In addition, Model 2 added neuroimaging data. The use of LASSO logistic regression allows for the identification of the strongest predictors among a large number of predictors.

**Results:**

Model 1 suggested that greater hostility, guilt, and negative urgency predicted higher likelihood of belonging to the high-risk group, whereas greater difficulty with abstract thinking predicted lower likelihood of belonging to the high-risk group. Model 2 suggested that greater hostility predicted higher likelihood of high-risk, whereas greater anterior cingulate thickness and increased activity in six brain regions—right superior frontal gyrus, left superior medial frontal gyrus, left superior frontal gyrus, right middle frontal gyrus, and right middle cingulate gyrus—predicted a lower likelihood of high SIB.

**Discussion:**

Negative urgency and SSD symptoms can classify high-and low-risk groups accurately, and inclusion of brain data slightly increased predictive accuracy.

## 1 Introduction

People with schizophrenia spectrum disorders (SSDs) have an elevated risk for suicidal ideation and behaviors (SIBs), with rates ranging from 4.9 to 22.3%, and comparable to those of individuals with depressive disorders (1–4). Recognized correlates of suicide risk in schizophrenia include several static and dynamic factors such as demographics, life events, a history of past suicide attempts, symptom severity, acute stressors, substance misuse, and social isolation (5, 6).

Studies have also shown that impulsivity may play some role in suicidality among individuals with SSDs (7–10). One dimension of impulsivity known as negative urgency has a unique relationship with suicidality in multiple populations (11–16). Negative urgency is a facet of impulsivity which describes a tendency to act impulsively in response to strong negative emotions (17, 18). Recently, Hoptman et al. (2023) provided initial evidence for a relationship between negative urgency and suicidal ideation and lifetime suicide attempt in individuals with SSDs (1).

Functional magnetic resonance imaging (fMRI) studies have implicated abnormal connectivity between prefrontal cortical regions including dorsolateral, medial, anterior cingulate, and orbitofrontal regions and subcortical structures (e.g., the amygdala and insula) with increased negative urgency in SSD (19–22). Most of these studies sought to link the neural circuitry of negative urgency with emotion abnormalities, substance abuse, aggressive attitudes, and violence in SSD.

In contrast, the relationship between negative urgency and suicidal ideation and behaviors in individuals with SSDs is largely unexplored. As an exception, Hoptman et al. (2024) utilized fMRI data to examine activation in areas of the brain of individuals with SSDs while they completed a task designed to evoke implicit regulation of negative affect and serve as a proxy task-based measure of negative urgency (1). They then compared the results of individuals with high levels of SIBs to individuals with low levels of suicidal ideation and behavior. They found lower activation patterns in several frontal regions including the DLPFC, medial frontal, and cingulate regions in individuals with SSDs and who endorse high SIBs. They also found inverse associations between some of these regions and negative urgency. The findings suggest overlap of activation in neural circuits implicated in negative urgency and SIB in individuals with SSDs. Their findings also raise the question of whether negative urgency and its circuitry can enhance the discrimination of high versus low SIBs in individuals with SSDs (1). Of note, empirical research suggests that higher cognitive ability is associated with higher SIBs in individuals with SSDs (23–25). This association is important to note, as it challenges assumptions that cognitive strengths are inherently protective and suggests that individuals with higher cognitive ability may be at elevated risk for more deliberate or concealed self-injurious behaviors.

Machine learning (ML) has been used in previous studies to identify risk factors for suicidality and has been found to be generally accurate (26). ML approaches are particularly useful for calibrating models with a relatively large number predictor variables in contrast to other analytical approaches (26–28). ML is an area within artificial intelligence (AI) that involves the use of algorithms that enable programs to “learn” from data automatically to improve. In healthcare and research, ML combined with large databases has the potential to allow for more specific and accurate diagnoses, treatments, and predictions (29). Specifically, least absolute shrinkage and selection operator (LASSO) logistic regression has been used in suicide research to identify risk factors and has been found to be more accurate than other ML models (30, 31). In a study by Bohaterwecisz and colleagues that utilized various ML models to identify suicide risk in a sample of individuals with schizophrenia, LASSO was found to reach the highest accuracy compared to other models when discriminating between suicide risk and non-suicide risk individuals using fMRI data (31).

The current study aims to examine the association between SSD symptoms, negative urgency, and SIBs via LASSO logistic regression. Because suicidal ideation and behavior in SSDs likely emerge from both psychological and neurobiological vulnerabilities, two LASSO models were tested to examine the relative and combined contributions of these domains. Model 1 captured behavioral and symptom-based predictors, while Model 2 integrated neuroimaging data to assess whether neural activity improved discrimination of high versus low SIB risk. Testing two models allows an assessment of the peak contribution of neuroimaging variables relative to established behavioral and symptom-level correlates of SIB. Based on previous evidence that negative urgency is associated with SIBs, the expectation of the current study is that the combination of statistical models and brain imaging data will provide additional evidence for the relationship between negative urgency and SIBs in individuals with SSDs.

## 2 Methods

### 2.1 Participants

Clinical data for this study was provided by participants from a previous study (13). The sample consisted of 35 individuals with a diagnosed SSD who were recruited from Rockland Psychiatric Center and/or referred by other researchers. All participants were between the ages of 18 and 60 and had capacity to sign written informed consent. The diagnosis of SSD was confirmed using the Mini International Neuropsychiatric Interview, v. 7.0.2. (M.I.N.I.) (32) or the Structural Clinical Interview for the DSM-5 (SCID-5) (33). The study consisted of both individuals in outpatient treatment and inpatient treatment. Imaging was obtained from 30 out of 35 participants due to issues regarding the process of imaging (i.e. inability to tolerate scanning, difficulty hearing task) or issues with the data following scanning. Individuals with a diagnosis of a substance use disorder in the past 3 months were excluded from the study. Participants met criteria for the study based on past-year SIB, or the endorsement of at least two suicide attempts in their lifetime. Participants who scored between the two SIB groups were excluded from the study.

### 2.2 Measures

Demographic variable information (i.e., age and sex) were collected for the study. Number count and severity of childhood trauma were also collected. Psychotic symptoms were measured by the Positive and Negative Syndrome Scale (PANSS) (34, 35). Premorbid intellectual ability was operationalized using the scaled score from the Wide Range Achievement Test- Version 4 (WRAT-4) (36, 37). Negative Urgency was measured using the full Urgency, Premeditation, Perseverance, Sensation Seeking, Positive Urgency scale (UPPS-P) (17, 19), which is comprised of five subscales: positive urgency, negative urgency, lack of premeditation, lack of perseverance, and sensation seeking. The Beck Scale for Suicidal Ideation (BSSI) (38) was used to assess past-week suicidal ideation and attempts. The Columbia Suicide Severity Rating Scale (C-SSRS) (39) was used to assess past year and lifetime SIBs. Both past-year and lifetime SIB were assessed using the C-SSRS. Based on scores from the C-SSRS, participants were classified into either a high SIB group or a low SIB group, and group membership was used as the outcome variable for the study. Individuals with a score of less than 2 lifetime SIBs were considered low SIB, and individuals with a score of greater than 3 in the last 12 months and/or at least two lifetime suicide attempts were considered high SIB. The past year criteria cutoffs are from suicidal ideation only.

#### 2.2.1 Brain imaging data

Imaging for the study was conducted via fMRI on individuals performing a task. Imaging took place at The Nathan S. Kline Institute for Psychiatric Research’s Center for Biomedical Imaging and Neuromodulation using a Siemens 3T TiM Trio and a 32-channel head coil. An anatomical scan was collected to provide cortical thickness measurements and to allow intersubject registration of functional images. The task involved implicitly manipulating emotional regulation in response to being shown affective pictures using neutral and negative sentences preceded by spoken statements (39, 40). During the task, participants viewed negative and neutral pictures from the International Affective Pictures System for three seconds each, which were preceded by either a spoken neutral statement or a spoken negative statement presented over a seven second window. Participants then were asked to rate the unpleasantness of the picture using the Self-Assessment Manikin (IAPS) (41). The task contrast was Negative pictures preceded by Neutral statements compared to Neutral pictures preceded by Neutral statements. Processing included motion correction, projection into standard space, scaling to a value of 100, smoothing with a 4mm Gaussian kernel, and regressing motion parameters (and their first derivatives), as well as white matter signal and CSF signal. Cluster names were identified using the AFNI N27-MNI atlas. Cluster size was determined using threshold-free cluster enhancement. Freesurfer version 5.3 Beta served to process and analyze acquired anatomical data and cortical thickness was estimated using Fischl and Dale’s method (42). The imaging data was processed using AFNI (43). Further details are reported in Hoptman et al. (2024) (1).

## 3 Statistical analysis

A least absolute shrinkage and selection operator (LASSO) logistic regression served to classify individuals with high and low suicidal ideation and behaviors (44). LASSO is a form of regularization which reduces errors caused by overfitting on data and multicollinearity by applying a penalty to the residual sum of squares to reduce the coefficients of less important predictors to zero (45, 46). LASSO is well suited for high-dimensional data in which the sample size may be similar or lower than the number of predictor variables. To select the optimal shrinkage parameters (i.e., lambda) for the LASSO models and obtain mean cross-validation estimates of model performance, a five-fold cross-validation with three repetitions was performed using the caret package (47). Splitting the sample into a calibration and cross-validation set makes it difficult to accurately characterize uncertainty in prediction. This is especially the case with small sample sizes. However, the use of repeated k-fold cross-validation can provide more reasonable estimates of how accurate the resultant model’s prediction would be in predicting unseen future data sets.

We implemented a nested cross-validation (i.e., outer/inner cross-validation framework) as follows. In each outer fold: (1) we split the outer-training data into 5 folds and repeated this 3 times; (2) for each candidate λ, we trained on the inner-training portion and computed ROC AUC on the inner-validation portion; (3) we averaged ROC AUC over the 15 inner resamples and chose the λ with the highest average; (4) we refit the model with that λ on the entire outer-training set and predicted the outer-test set. We then combined all outer-test predictions to estimate performance. All predictors were centered and scaled within each training split of the nested cross-validation to avoid leakage. If a variable had missing values, we applied k-nearest-neighbors imputation (k=5) on the training split only. The LASSO penalty (lambda) was tuned by maximizing AUC on the inner-training split. The model was refit on the outer-training split and then evaluated on the held-out test split. Tuning was performed independently for every outer split and for each model.

The performance metric of the models is Area under the Receiver Operating Characteristic Curve (AUC), which can be classified into five categories: fail (.50–.59), poor (.60–.69), fair (.70–.79), good (.80–.89), and excellent (.90–1.00) (48). In order to capture the variability of each model’s performance across the 15 cross-validations, we calculated and reported a 95% bias-corrected and accelerated confidence interval of the AUCs using 5,000 bootstrapped resamples in order to reduce the risk of overfitting and to provide a more complete picture of model performance, in addition to also reporting sensitivity, specificity, negative predictive value (NPV), positive predictive value (PPV), and their respective confidence intervals. The resultant optimal lambda parameters were used to refit the models to obtain the predictor coefficients needed to rank relative importance of predictors in each model. Specifically, variable importance was estimated by reordering the coefficients of predictors whose values are not shrunken to zero on a scale from 0 to 100 with the predictor of the greatest magnitude scoring 100.

The data were analyzed in two models. Model 1 included demographic variables (i.e., age and sex), number count and severity of childhood trauma, ratings on all 30 PANSS items, WRAT-4 estimated premorbid intellectual ability, and the UPPS-P urgency subdomains. Model 2 included Model 1 variables, along with the addition of structural and task-related fMRI brain imaging data. The models were then compared using a paired difference in AUC (ΔAUC) to adjudge the incremental predictive validity of adding imaging variables to the prediction of SIB risk. A comparison of model predictive properties is shown in **Table 2**.

## 4 Results

**Table 1** summarizes the demographic and clinical characteristics of the study sample and a univariate comparison of the high and low SIB groups. There were statistically significant differences between both groups in past year and lifetime suicidal ideation, lifetime attempts, negative urgency, and the PANSS depression subscale. There were no differences on any other demographic or clinical variables. Although the high-risk group endorsed between 1 and 8 lifetime attempts (see **Supplementary Figure S1**), none of the participants endorsed a suicide attempt in the past year.

**Table 1:** Demographic and clinical characteristics of the neuroimaging sample.

Variables	Total sample (n=30)	Low SIB (n=16)	High SIB (n=14)
Age (years)	41.07 (10.95)	40.12 (11.02)	42.14 (10.95)
Sex (M/F)	26/4	14/2	12/2
Education (years)^1^	4.07 (1.05)	3.94 (1.81)	4.21 (0.89)
Handedness (RH/LH)^2^	26/4	14/2	12/2
CPZ Equivalents (mg)^3^	1167.25 (734.08)	999.97 (836.30)	1358.41 (566.83)
C-SSRS SI (life)	2.33 (2.42)	0.12 (.34)	4.856 (3.63)**
C-SSRS Attempts (life)	1 [0,8]	0 [0,0]	3.57 (2.13)**
C-SSRS SI (past year)	0.70 (1.08)	0.12 (0.34)	1.36 (1.27)**
C-SSRS Attempts (past year)	0 [0,0]	0 (0)	0 (0)
BSSI Suicidal Ideation	2.33 (3.93)	0.50 (2.0)	4.43 (4.59)*
Negative Urgency (total)	27.13 (8.84)	23.0 (8.0)	31.86 (7.44)*
Positive Urgency (total)	28.20 (10.42)	26.5 (10.11)	30.14 (10.81)
PANSS Negative Scale	16.20 (7.21)	17.81 (8.65)	14.36 (4.78)
PANSS Excitement Scale	9.73 (3.15)	8.81 (3.04)	10.78 (3.04)
PANSS Cognitive Scale	12.80 (3.76)	13.81 (3.5)	11.64 (3.83)
PANSS Positive Scale	12.0 (4.88)	10.87 (4.94)	13.28 (4.66)
PANSS Depression Scale	12.10 (5.20)	9.75 (4.74)	14.78 (4.46)*

CPZ equivalents (Woods, 2003); C-SSRS, Columbia-Suicide Severity Rating Scale (Posner et al., 2011); LH, Left-handed; RH, right-handed; BSSI, Beck Scale for Suicidal Ideation (Beck et al., 1979); PANSS, Positive and Negative Syndrome Scale (Kay et al., 1987; Lindenmayer et al.,1994); ^1^Descriptive statistics are mean and standard deviation (SD) or median and [minimum, maximum]. ^1^Education scores correspond to the Hollingshead Education Scale and range from 0-7(0=N/a, 1=<7th grade, 2=Junior high school, 3=Partial high school, 4=High school graduate, 5=Partial college, 6=College graduate, 7=Graduate/Professional training). *=Significant at p<.05. **=Significant at p<.001.

### 4.1 Discrimination of LASSO models

#### 4.1.1 Model 1

**Figure 1:**
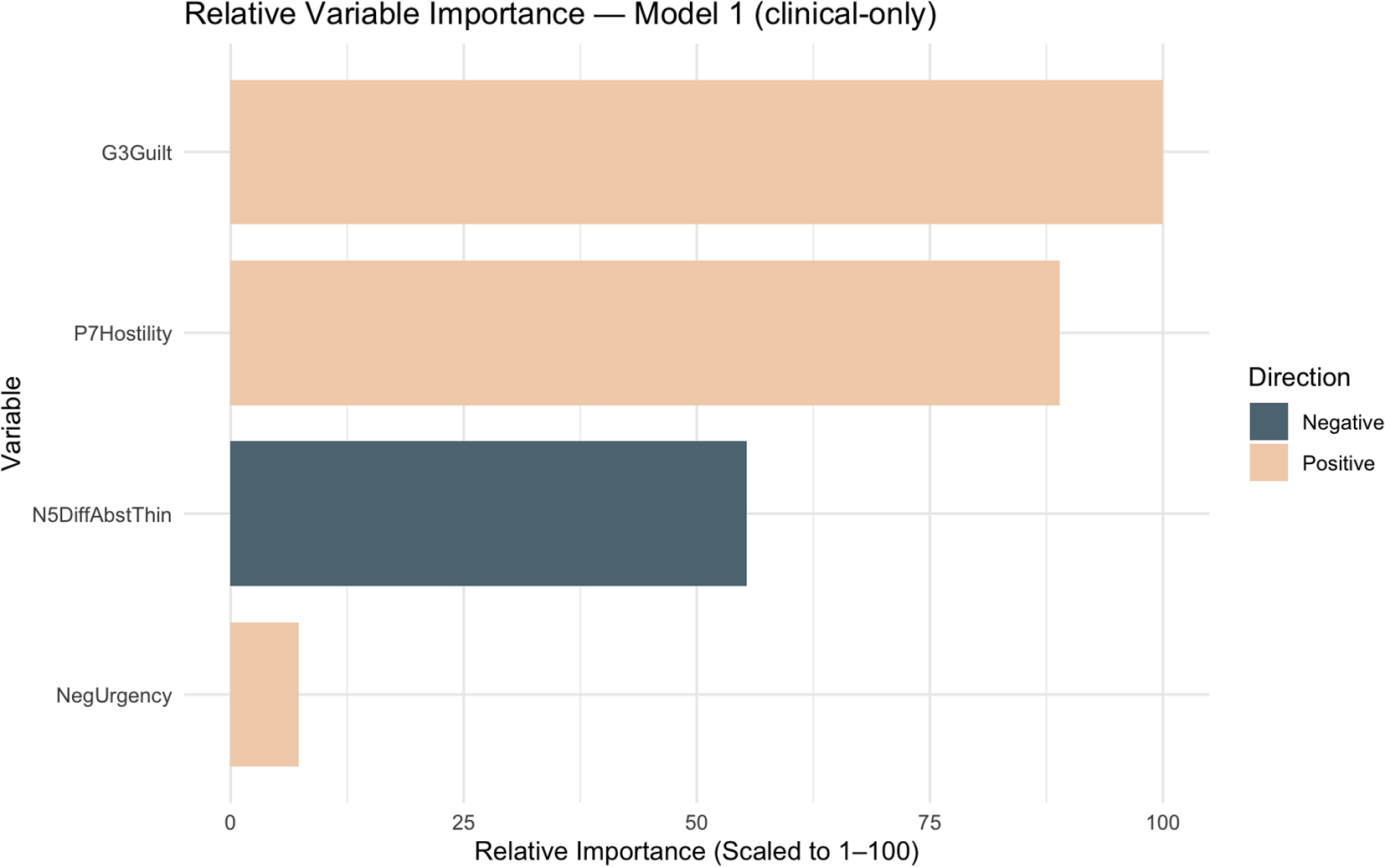
Relative variable importance predicting high SIB group in model 1. G3Guilt, G3 Guilt Feelings; P7Hostility, P7 Hostility; N5DiffAbstrThin, N5 Difficulty in Abstract Thinking; NegUrgency, Negative Urgency.

Designating membership in the high SIB group as the outcome variable, Model 1 demonstrated good predictive accuracy, with an outer-loop AUC of 0.732 (95% CI [0.538–0.926], DeLong), and an Area Under Precision-Recall Curve (AUPRC) of 0.653 (See **Table 2**). Using a 0.50 decision threshold, the model achieved an accuracy of 0.767, sensitivity of 0.643, specificity of 0.875, positive predictive value (PPV) of 0.818, and negative predictive value (NPV) of 0.737. **Figure 1** presents the variables ranked in descending order of importance. Greater hostility, guilt, and negative urgency predicted a higher likelihood of belonging to the high SIB group, whereas greater difficulty with abstract thinking predicted a lower likelihood of high SIB.

#### 4.1.2 Model 2

Designating membership in the high SIB group as the outcome variable, Model 2 was estimated with additional features and evaluated using nested cross-validation. For each possible λ, the model was trained on the inner-training set and evaluated using ROC AUC on the inner-validation set. The λ yielding the highest mean inner-validation AUC was selected, and the final model was retrained on the complete outer-training set and tested on the outer-test set.

Performance was summarized from all outer-test predictions. Discrimination was strong, with an outer-loop AUC of 0.893 (95% CI [0.779, 1.000], DeLong) and an AUPRC of 0.896, indicating high predictive accuracy under moderate class prevalence. Using a 0.50 decision threshold, the model achieved an accuracy of 0.733, sensitivity of 0.857, specificity of 0.625, positive predictive value (PPV) of 0.667, and negative predictive value (NPV) of 0.833.

**Figure 2:**
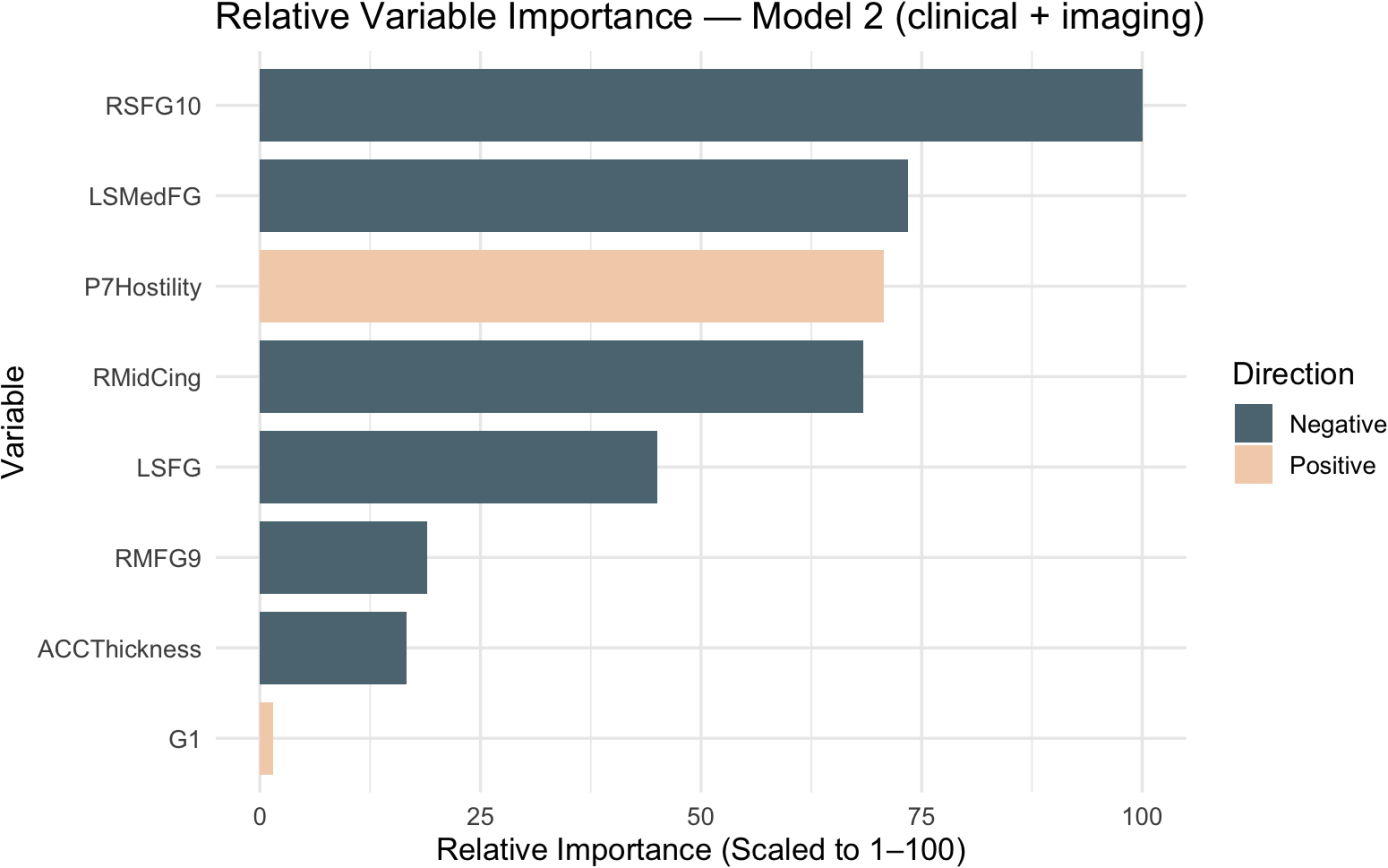
Relative variable importance predicting high SIB group in model 2. LSMedFG= Left Superior Middle Frontal Gyrus; RMidCing =Right Middle Cingulate Gyrus; LSFG = Left Superior Frontal Gyrus; RSFG10 = Right Superior Frontal Gyrus; RSTG = Right Superior Temporal Gyrus; RrACC = Right rostral Anterior Cingulate Cortex; RMFG9 = Right Middle Frontal Gyrus; LMedFG9 = Left Middle Frontal Gyrus.

**Figure 2** displays the variables ranked by importance (See **Table 2**). Greater hostility predicted a higher likelihood of high SIB, whereas anterior cingulate thickness and increased activity in six brain regions—right superior frontal gyrus, left superior medial frontal gyrus, left superior frontal gyrus, right middle frontal gyrus, and right middle cingulate gyrus—predicted a lower likelihood of high SIB.

### 4.2 Calibration analysis and comparison of model performance

**Figure 3:**
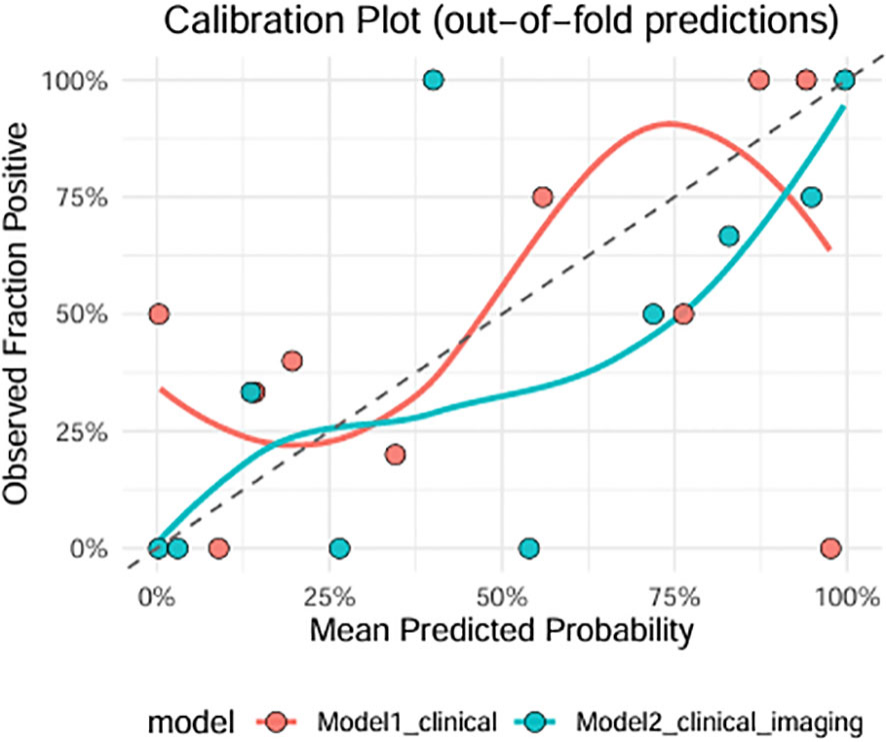
Calibration plots for Model1 and Model 2 predicting high SIB.

**Figure 3** depicts the calibration plot of out-of-fold predictions with both Model 1 and Model 2 superimposed on the ideal 45° reference line. Model 2 demonstrated a closer alignment to the reference line across the probability spectrum, especially in the mid-range of predicted probabilities. A calibration analysis of Model 2 produced a slope of 0.66 and an intercept of −0.53, suggesting that the model tended to underestimate the probability of high SIB, particularly among individuals at higher predicted risk. Model 1 showed greater downward deviation at higher predicted risk levels, indicating stronger underestimation of true risk. These results suggest that although both models discriminate high and low SIB risk well, Model 2 provides more reliable probability estimates, yielding improved risk stratification but tending to conservatively estimate risk. It is noteworthy however that Model 2 retained high sensitivity which may be preferable in suicide risk prediction.

**Table 2:** Model performance of lasso logistic regression predicting high low SIB group.

Performance metrics	Model 1	Model 2
AUC	0.73 [0.54, 0.93]	0.89 [0.78, 1.00]
Sensitivity	0.64 [0.35, 0.87]	0.86 [0.57, 0.98]
Specificity	0.88 [0.62, 0.98]	0.62 [0.35, 0.85]
NPV	0.74 [0.49, 0.91]	0.83 [0.52, 0.98]
PPV	0.82 [0.48, 0.98]	0.67 [0.41, 0.87]

AUC, area under the ROC curve; 95% CI, 95% confidence interval; NPV, negative predictive value; PPV, positive predictive value. Lambda parameters were identified through five−fold cross−validation repeated three times for lasso models.

To evaluate whether inclusion of neuroimaging predictors improved classification accuracy, we compared Model 2 to Model 1 using a paired difference in ΔAUC. The analysis yielded a ΔAUC of 0.161 (bootstrap 95% CI [0.038, 0.306]), indicating a modest improvement in discrimination when neuroimaging features were added. Because the 95% CI did not include zero, this indicates a statistically reliable improvement in discrimination when neuroimaging predictors were added. Moreover, the direction of the effect suggests a potential enhancement in classification performance associated with the inclusion of neuroimaging variables.

### 4.3 Comparison to simple baseline models

To contextualize the performance of the LASSO models, we compared them to two simple baseline models trained using single predictors—negative urgency and hostility—under the same repeated 5-fold cross-validation procedure. Performance was summarized from the average out-of-fold predictions, and 95% confidence intervals were computed using the DeLong method.

The Negative Urgency–only model achieved an AUC of 0.748 (95% CI [0.640, 0.856]), while the Hostility–only model achieved an AUC of 0.838 (95% CI [0.746, 0.929]). Both LASSO models outperformed these baselines, demonstrating that combining multiple behavioral and neuroimaging predictors improved discrimination beyond the contribution of any single variable.

These results highlight the incremental predictive value of multivariable modeling and suggest that while hostility alone offers relatively strong discrimination, incorporating additional features—particularly neuroimaging measures—further enhances classification performance for identifying individuals at high risk of SIB.

## 5 Discussion

LASSO logistic regression was found to show good predictive accuracy for both Model 1 and Model 2 in distinguishing high versus low SIB in people with SSDs. In Model 1, greater hostility, guilt, and negative urgency were associated with higher likelihood of high SIB, whereas greater difficulty with abstract thinking was associated with lower likelihood of high SIB. Model 2 retained hostility as a prioritized variable associated with higher likelihood of high SIB, while increased anterior cingulate thickness and greater activity in the right superior frontal gyrus, left superior medial frontal gyrus, left superior frontal gyrus, right middle frontal gyrus, and right middle cingulate gyrus were associated with lower likelihood of high SIB.

In Model 1, the most influential predictors included both clinically-rated symptoms (e.g. hostility, guilt, difficulty with abstract thinking), and trait urgency, suggesting that emotional impulsivity plays an important role in SIB even when accounting for symptom severity among individuals with SSDs. The identification of negative urgency as a risk factor associated with higher likelihood of high SIB is consistent with previous findings linking negative urgency to suicide risk and prediction of suicidal ideation (11, 14).

Negative urgency, hostility, and guilt each reflect heightened sensitivity to negative affective states combined with difficulties regulating emotional responses. Negative urgency is a tendency to act impulsively in the context of emotional distress; hostility reflects externally directed negative affect; and guilt represents internally directed negative affect often linked with self-blame and ruminative tendencies. Together, these constructs suggest that individuals who experience intense emotional distress and lack effective emotion regulation strategies may be at elevated risk for SIB (49, 50). Conversely, difficulty with abstract thinking—the only behavioral variable associated with lower likelihood of SIB—may indicate reduced capacity for complex self-referential or future-oriented thought (23, 51). Individuals with pronounced abstraction deficits may be less able to engage in reflective negative self-appraisals, future-oriented suicidal planning, or cognitively mediated forms of self-directed blame (24, 51). This interpretation aligns with theories suggesting that certain cognitive deficits in SSD may, paradoxically, reduce engagement in the kinds of internal cognitive-emotional processes that contribute to SIB (23–25, 51–53).

A broader literature suggests that negative affectivity is highly prevalent in SSD and is often linked to cognitive control deficits (54–56). Heightened emotional reactivity could drive negative urgency by overwhelming the cognitive resources required to consider interpersonal goals, anticipate consequences, inhibit maladaptive impulses, and deploy adaptive coping strategies (57–60). Prior research has associated diminished cognitive control with maladaptive behaviors like impulsive aggression in SSD. The current study extends this pattern to SIB. Together, these predictors suggest that SIB risk in SSD may emerge from a convergence of heightened negative emotional reactivity and weakened top-down regulatory mechanisms, whereas low-risk may reflect preserved engagement of the prefrontal-cingulate regulatory circuitry that supports emotion regulation and impulse control.

The finding that increased activity in several cortical regions was associated with reduced likelihood of high SIB–although novel–is supported by prior research indicating that variations in brain activity are associated with suicide risk in SSD (31, 61). The brain regions highlighted by Model 2 further underscore the interplay between affective instability and cognitive control to SIB risk in SSD and provides insight into potential protective neural mechanisms. The left and right superior and middle frontal gyri are core regions in the dorsolateral prefrontal cortex (DLPFC). They play a central role in executive control, cognitive reappraisal, self-referential thought, top-down modulation of emotional responses, and inhibition of maladaptive responses (58–62). Dysfunction in the DLPFC has been linked to SIB transdiagnostically (61).

These may further interact with other regions (e.g., right superior temporal gyrus), to support social cognition and interpretation of emotional cues, which may buffer against interpersonal misunderstandings that trigger affective dysregulation and self-directed aggression (62, 63). The right middle cingulate gyrus (MCG) integrates cognitive and emotional information and plays a central role in conflict monitoring, interoceptive awareness, and affect regulation (58, 59). Increased activation in these circuits in the low-SIB group may indicate stronger engagement of top-down regulatory networks that mitigate dysregulated affect and inhibit impulsive responses, by integrating emotional signals with contextual information. These neural findings complement the behavioral predictors, collectively supporting a model in which risk for SIB in SSD arises from impaired coordination between emotional arousal and cognitive control, whereas resilience is supported by strengthened regulatory neural circuitry.

These findings are consistent with prior studies using fMRI showing that greater activity in prefrontal and cingulate activation is associated with resilience to suicidal behavior and better emotion regulation capacity in healthy and clinical groups (31). Conversely, hypoactivation in these same regions has been repeatedly observed among individuals with high suicidality, particularly during tasks involving cognitive interference or emotional conflict. Overall, the pattern of findings points toward a convergent mechanism in which impaired cognitive regulation of negative emotion increases risk for SIB, while heightened engagement of regulatory frontal–cingulate–temporal networks confers protection.

Beyond circuit-level explanations, emerging neurobiological models suggest that the maintenance of these protective neural functions may depend on global homeostatic systems that support metabolic and synaptic efficiency (64, 65). The glymphatic system—a brain-wide perivascular network that facilitates the clearance of metabolic waste and neurotoxic solutes—has been increasingly recognized as essential for maintaining neuronal health and network integrity. Disruption of glymphatic clearance has been linked to neuroinflammation and reduced functional connectivity within prefrontal and cingulate regions implicated in emotion regulation (66). Thus, intact glymphatic function may indirectly support the neural efficiency and resilience observed in the low-SIB group, promoting recovery and stability in circuits that modulate affect and impulse control.

The present study represents one of the first to use machine learning to jointly examine clinical, cognitive, and neuroimaging predictors of SIB in individuals with SSDs. The results of this study contribute to the growing literature data-driven approaches to suicide risk prediction and highlight the potential roles of emotion-based impulsivity, negative affect, and neural regulatory mechanisms as meaningful predictors of SIB. Although Model 2 tended to slightly underestimate predicted risk, this pattern should be interpreted within the clinical decision-making context. In suicide risk assessment, both over-estimation and under-estimation impose a cost—over-estimation may increase false positives and burden clinical resources, whereas under-estimation risks failure to identify individuals in acute danger. In the current study, Model 2 retained high sensitivity across thresholds, indicating that risk under-estimation did not come at the cost of missing high-risk individuals. Thus, while Model 2 provides a reliable ranking of relative risk, the threshold for high-SIB classification can be adjusted based on clinical setting and tolerance for false positives versus false negatives.

Future research should prioritize replication in larger independent samples and longitudinal designs to test dynamic changes in cognitive-emotional regulation and neural engagement over time. Longitudinal studies integrating ecological momentary assessment and multimodal imaging could further clarify how dynamic interactions between neural regulation and negative urgency confer risk or resilience for SIB.

### 5.1 Limitations

The current study has limitations. First, the study sample is relatively small, which may limit the interpretability of the results of the model. Second, ROIs were derived from the same data used for modeling, so it is possible that the data reflects issues of double-dipping. Third, there was no healthy control group or psychiatric control group in the study, which limits the ability to conclude that the findings are specific to an SSD population. Additionally, the study participants were all individuals with chronic, severe mental illness and were on antipsychotic medication, further limiting the generalizability of the findings to individuals at different stages of illness. Fourth, the study did not include a broad measure of global cognitive ability, which would have provided the authors with a more comprehensive understanding of the cognitive abilities of the participants than what was included in the study. These limitations underscore the need for fine-grained, temporally sensitive evaluations of suicidal ideation and related behaviors, which may fluctuate rapidly and vary across the course of illness.

## 6 Conclusions

In conclusion, LASSO logistic regression as a ML model was found to show good predictive accuracy when identifying risk factors for high and low SIB. The first model identified greater hostility, guilt, and negative urgency as predictive of higher likelihood of high SIB when examining demographic factors, cognitive factors, and clinical factors, and the second model identified greater hostility as predictive of higher likelihood of higher SIB when adding structural and brain imaging data to the model. Difficulty with abstract thinking and increased activity in certain brain regions predicted lower likelihood of high SIB. The inclusion of brain imaging data increased the predictive accuracy of the model and contributes to the novelty of the study.

## Data Availability

The raw data supporting the conclusions of this article will be made available by the authors, without undue reservation.
