# Supplementary material for: Circuitry correlates of negative urgency and suicidality in schizophrenia spectrum disorders: a LASSO regression study
Source: Front Psychiatry. 2025 Dec 9;16:1678555. doi: 10.3389/fpsyt.2025.1678555 (PMC12722959; doi:10.3389/fpsyt.2025.1678555)
Supplement: Supplementary file 1 [file DataSheet1.docx]

Supplement To: Circuitry Correlates of Negative Urgency and Suicidality in Schizophrenia Spectrum Disorders: A LASSO Regression Study

Tyler Pia^!1^, Enna Sanghvi^!1^, Mark Shuquan Chen^2^, Thomas Kim^1^, Matthew J. Hoptman^3,4^, Anthony O. Ahmed^1*^

^1^Department of Psychiatry, Weill Cornell Medicine | NewYork-Presbyterian – Westchester

^2^Department of Psychology, Yale University

^3^Division of Clinical Research, The Nathan S. Kline Institute for Psychiatric Research

^4^Department of Psychiatry, NYU Grossman School of Medicine


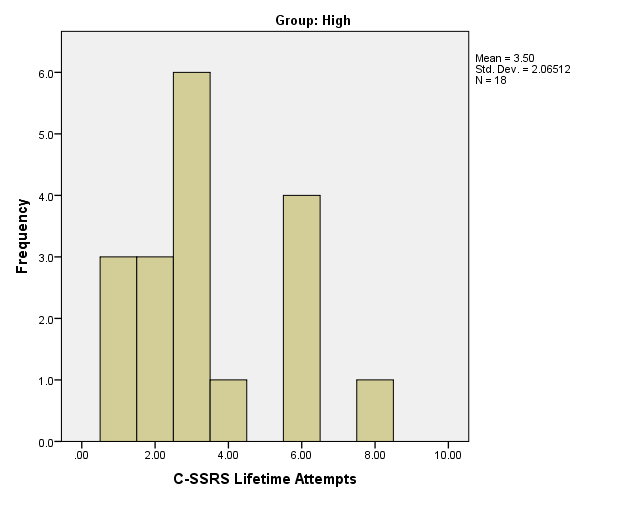


*Supplemental Figure S1. Distribution of Lifetime Suicide Attempts in the High SIB Group*


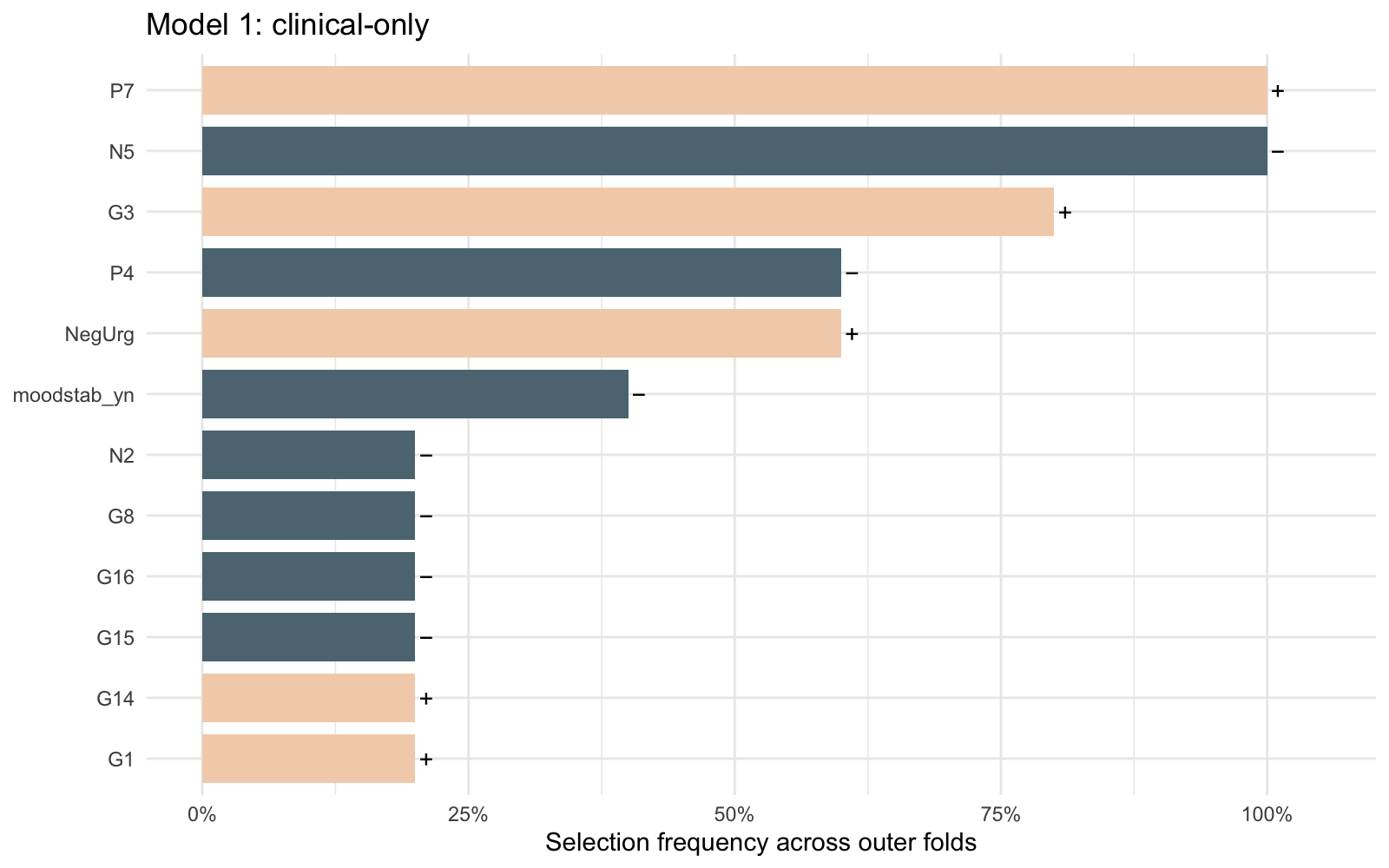


*Supplemental Figure S2. Selection Frequencies of Prioritized Variables Predicting High SIB Group in Model 1.*


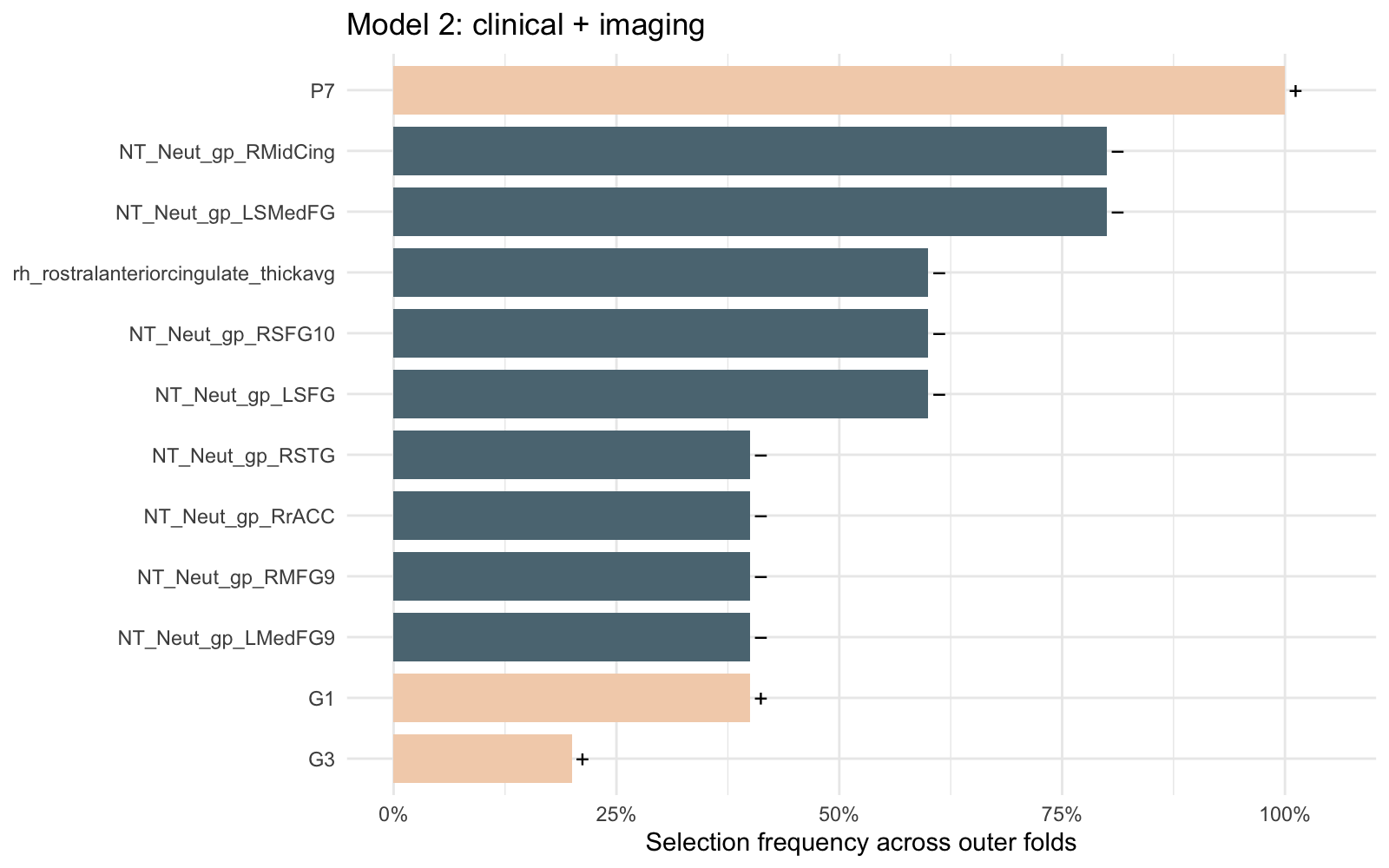


*Supplemental Figure S3. Selection Frequencies of Prioritized Variables Predicting High SIB Group in Model 2.* LSMedFG= Left Superior Middle Frontal Gyrus; RMidCing =Right Middle Cingulate Gyrus; LSFG = Left Superior Frontal Gyrus; RSFG10 = Right Superior Frontal Gyrus; RSTG = Right Superior Temporal Gyrus; RrACC = Right rostral Anterior Cingulate Cortex; RMFG9 = Right Middle Frontal Gyrus; LMedFG9 = Left Middle Frontal Gyrus

Supplemental Table S1. TRIPOD Checklist of Adherence to Reporting Standards

| **Section/Topic** | **Item** | **Checklist Item** | **Page** |
| --- | --- | --- | --- |
| **Title and abstract** | | | |
| Title | 1 | Identify the study as developing and/or validating a multivariable prediction model, the target population, and the outcome to be predicted. | 1 |
| Abstract | 2 | Provide a summary of objectives, study design, setting, participants, sample size, predictors, outcome, statistical analysis, results, and conclusions. | 1-2 |
| **Introduction** | | | |
| Background and objectives | 3a | Explain the medical context (including whether diagnostic or prognostic) and rationale for developing or validating the multivariable prediction model, including references to existing models. | 2-3 |
|  | 3b | Specify the objectives, including whether the study describes the development or validation of the model or both. | 3 |
| **Methods** | | | |
| Source of data | 4a | Describe the study design or source of data (e.g., randomized trial, cohort, or registry data), separately for the development and validation data sets, if applicable. | 3 |
|  | 4b | Specify the key study dates, including start of accrual; end of accrual; and, if applicable, end of follow-up. |  |
| Participants | 5a | Specify key elements of the study setting (e.g., primary care, secondary care, general population) including number and location of centres. | 3 |
|  | 5b | Describe eligibility criteria for participants. | 3 |
|  | 5c | Give details of treatments received, if relevant. | NA |
| Outcome | 6a | Clearly define the outcome that is predicted by the prediction model, including how and when assessed. | 4 |
|  | 6b | Report any actions to blind assessment of the outcome to be predicted. | NA |
| Predictors | 7a | Clearly define all predictors used in developing or validating the multivariable prediction model, including how and when they were measured. | 3-4 |
|  | 7b | Report any actions to blind assessment of predictors for the outcome and other predictors. | NA |
| Sample size | 8 | Explain how the study size was arrived at. | 3 |
| Missing data | 9 | Describe how missing data were handled (e.g., complete-case analysis, single imputation, multiple imputation) with details of any imputation method. | 3 |
| Statistical analysis methods | 10a | Describe how predictors were handled in the analyses. | 5 |
|  | 10b | Specify type of model, all model-building procedures (including any predictor selection), and method for internal validation. | 4-5 |
|  | 10d | Specify all measures used to assess model performance and, if relevant, to compare multiple models. | 6 |
| Risk groups | 11 | Provide details on how risk groups were created, if done. | NA |
| **Results** | | | |
| Participants | 13a | Describe the flow of participants through the study, including the number of participants with and without the outcome and, if applicable, a summary of the follow-up time. A diagram may be helpful. | NA |
|  | 13b | Describe the characteristics of the participants (basic demographics, clinical features, available predictors), including the number of participants with missing data for predictors and outcome. | 15 (Table 1) |
| Model development | 14a | Specify the number of participants and outcome events in each analysis. | 4 |
|  | 14b | If done, report the unadjusted association between each candidate predictor and outcome. | NA |
| Model specification | 15a | Present the full prediction model to allow predictions for individuals (i.e., all regression coefficients, and model intercept or baseline survival at a given time point). | NA |
|  | 15b | Explain how to the use the prediction model. | 5 |
| Model performance | 16 | Report performance measures (with CIs) for the prediction model. | 5-6 |
| **Discussion** | | | |
| Limitations | 18 | Discuss any limitations of the study (such as nonrepresentative sample, few events per predictor, missing data). | 9 |
| Interpretation | 19b | Give an overall interpretation of the results, considering objectives, limitations, and results from similar studies, and other relevant evidence. | 7-8 |
| Implications | 20 | Discuss the potential clinical use of the model and implications for future research. | 7-8 |
| **Other information** | | | |
| Supplementary information | 21 | Provide information about the availability of supplementary resources, such as study protocol, Web calculator, and data sets. | 9 |
| Funding | 22 | Give the source of funding and the role of the funders for the present study. | 10 |

# Machine Learning Predicting Suicide - Schizophrenia

library(dplyr)

library(haven)

library(stringr)

library(tibble)

library(ggplot2)

library(scales) # rescale()

library(caret)

library(glmnet)

library(boot) # bootstrap CIs

library(caret)

library(pROC)

library(tibble)

dat0 <- read_sav()

safe_pick <- function(nms, pool) nms[nms %in% pool][1]

safe_multi_rename <- function(df, mapping_list) {

for (new_nm in names(mapping_list)) {

candidates <- mapping_list[[new_nm]]

src <- safe_pick(candidates, names(df))

if (!is.na(src) && !is.null(src)) {

df <- dplyr::rename(df, !!new_nm := dplyr::all_of(src))

}

}

df

}

# KNN imputation wrapper:

impute_knn_df <- function(df, k = 5, scale_vars = TRUE) {

if (!anyNA(df)) return(df)

if (requireNamespace("DMwR2", quietly = TRUE)) {

return(DMwR2::knnImputation(as.data.frame(df), k = k, scale = scale_vars, meth = "weighAvg"))

} else if (requireNamespace("VIM", quietly = TRUE)) {

out <- VIM::kNN(df, k = k, imp_var = FALSE)

return(as.data.frame(out))

} else {

stop("Install one of: install.packages('DMwR2') or install.packages('VIM')")

}

}

# Find PANSS either like P1/N1/G1... or PAN0P1/PAN0N1/PAN0G1...

panss_cols <- unique(c(

grep("^PAN0(P|N|G)\\d{1,2}$", names(dat0), ignore.case = TRUE, value = TRUE),

grep("^(P|N|G)\\d{1,2}$", names(dat0), ignore.case = TRUE, value = TRUE)

))

base_candidates <- c(

"Group", "DEM001", "DEM002",

"CTQ_CHILD", "CTQ_CHILD_SEV",

"NegUrg", "PosUrg", "UPP_SS", "UPP_Premed", "UPP_persev",

"NT_Neut_gp_LSMedFG", "NT_Neut_gp_RrACC", "NT_Neut_gp_RMidCing",

"NT_Neut_gp_RSTG", "NT_Neut_gp_LSFG", "NT_Neut_gp_RMFG9", "NT_Neut_gp_RSFG10",

"NT_Neut_gp_RSFG9", "NT_Neut_gp_LMedFG9",

"rh_rostralanteriorcingulate_thickavg", "WRAT_SS",

"medtype", "cpzequiv", "moodstab_yn"

)

dat_sel <- dat0 %>%

dplyr::select(dplyr::any_of(base_candidates), dplyr::all_of(panss_cols))

rename_map <- list(

Suicide = c("Group"),

Age = c("DEM001","age","Age"),

Sex = c("DEM002","sex","Sex"),

Number_CTQ = c("CTQ_CHILD"),

Severity_CTQ = c("CTQ_CHILD_SEV"),

NegUrgency = c("NegUrg"),

PosUrgency = c("PosUrg"),

SensationSeek= c("UPP_SS"),

LackPremed = c("UPP_Premed"),

LackPerse = c("UPP_persev"),

LSMedFG = c("NT_Neut_gp_LSMedFG"),

RrACC = c("NT_Neut_gp_RrACC"),

RMidCing = c("NT_Neut_gp_RMidCing"),

RSTG = c("NT_Neut_gp_RSTG"),

LSFG = c("NT_Neut_gp_LSFG"),

RMFG9 = c("NT_Neut_gp_RMFG9"),

RSFG10 = c("NT_Neut_gp_RSFG10"),

RSFG9 = c("NT_Neut_gp_RSFG9"),

LMedFG9 = c("NT_Neut_gp_LMedFG9"),

ACCThickness = c("rh_rostralanteriorcingulate_thickavg"),

WRAT = c("WRAT_SS"),

# PANSS (support both naming styles)

P1Delu = c("PAN0P1","P1"),

P2ConcDiso = c("PAN0P2","P2"),

P3Hallu = c("PAN0P3","P3"),

P4Excite = c("PAN0P4","P4"),

P5Grandi = c("PAN0P5","P5"),

P6Suspicion = c("PAN0P6","P6"),

P7Hostility = c("PAN0P7","P7"),

N1Blunt = c("PAN0N1","N1"),

N2EmoWithdraw= c("PAN0N2","N2"),

N3PoorRapp = c("PAN0N3","N3"),

N4Apathy = c("PAN0N4","N4"),

N5DiffAbstThin = c("PAN0N5","N5"),

N6LackSpon = c("PAN0N6","N6"),

N7SterThin = c("PAN0N7","N7"),

G1Somatic = c("PAN0G1","G1"),

G2Anxiety = c("PAN0G2","G2"),

G3Guilt = c("PAN0G3","G3"),

G4Tension = c("PAN0G4","G4"),

G5Mannerism = c("PAN0G5","G5"),

G6Depres = c("PAN0G6","G6"),

G7MotoReta = c("PAN0G7","G7"),

G8Uncooper = c("PAN0G8","G8"),

G9UnusThou = c("PAN0G9","G9"),

G10Disorient = c("PAN0G10","G10"),

G11PoorAtte = c("PAN0G11","G11"),

G12LackJudg = c("PAN0G12","G12"),

G13DisturbVolition = c("PAN0G13","G13"),

G14PoorImpul = c("PAN0G14","G14"),

G15Preoccupation = c("PAN0G15","G15"),

G16SociAvoi = c("PAN0G16","G16")

)

dat <- safe_multi_rename(dat_sel, rename_map)

# Outcome

if (!"Suicide" %in% names(dat)) stop("Outcome column not found after renaming.")

if (inherits(dat$Suicide, "labelled")) dat$Suicide <- haven::as_factor(dat$Suicide)

if (is.numeric(dat$Suicide)) {

dat$Suicide <- factor(dat$Suicide, levels = c(1,0), labels = c("HighSIB","LowSIB"))

} else {

dat$Suicide <- factor(dat$Suicide)

levels(dat$Suicide) <- ifelse(grepl("1|high|sib|yes", levels(dat$Suicide), ignore.case = TRUE),

"HighSIB","LowSIB")

}

# Sex (only if present)

if ("Sex" %in% names(dat)) {

dat <- dat %>%

mutate(

Sex = if (inherits(Sex, "labelled")) haven::as_factor(Sex) else Sex,

Sex = dplyr::case_when(

is.numeric(Sex) & Sex == 1 ~ 1,

is.numeric(Sex) & Sex == 2 ~ 0,

is.character(Sex) & str_detect(str_to_lower(Sex), "^m") ~ 1,

is.character(Sex) & str_detect(str_to_lower(Sex), "^f") ~ 0,

TRUE ~ NA_real_

),

Sex = factor(Sex, levels = c(1,0), labels = c("Male","Female"))

)

}

# ---- 4) Impute predictors (KNN) ----

dat_impute <- subset(dat, select = -c(Suicide))

dat_DV <- subset(dat, select = c(Suicide))

dat_IV_impute <- impute_knn_df(dat_impute, k = 5, scale_vars = TRUE)

dat_final <- cbind(dat_DV, dat_IV_impute)

# Youden's J threshold from ROC on the TRAIN (inner) data

youden_threshold <- function(truth, prob_pos) {

roc_obj <- pROC::roc(response = truth, predictor = prob_pos, levels = c("LowSIB","HighSIB"), direction = "<")

as.numeric(pROC::coords(roc_obj, "best", ret = "threshold", best.method = "youden"))

}

# Outer/Inner (nested) CV for LASSO

set.seed(42)

y <- dat_final$Suicide

X <- dat_final %>% select(-Suicide)

# Stratified outer folds

K_OUTER <- 5

outer_folds <- createFolds(y, k = K_OUTER, returnTrain = FALSE)

# Inner CV control

inner_ctrl <- trainControl(

method = "repeatedcv",

number = 5, repeats = 3,

classProbs = TRUE,

summaryFunction = twoClassSummary,

savePredictions = "final"

)

lambda_grid <- expand.grid(.alpha = 1, .lambda = seq(0.001, 0.1, by = 0.001))

outer_preds <- vector("list", length(outer_folds))

outer_j_thresholds <- numeric(length(outer_folds))

outer_best_lambda <- numeric(length(outer_folds))

for (i in seq_along(outer_folds)) {

test_idx <- outer_folds[[i]]

train_idx <- setdiff(seq_len(nrow(dat_final)), test_idx)

dtrain <- dat_final[train_idx, , drop = FALSE]

dtest <- dat_final[test_idx, , drop = FALSE]

# INNER: tune lambda on dtrain

set.seed(100 + i)

mod_inner <- train(

Suicide ~ .,

data = dtrain,

method = "glmnet",

trControl = inner_ctrl,

tuneGrid = lambda_grid,

family = "binomial",

metric = "ROC",

preProcess = c("center","scale")

)

best_lambda <- mod_inner$bestTune$lambda

outer_best_lambda[i] <- best_lambda

# Compute Youden J threshold on the inner model using inner CV preds

inner_cv <- mod_inner$pred %>%

dplyr::filter(lambda == best_lambda) %>%

dplyr::mutate(obs = factor(obs, levels = c("LowSIB","HighSIB")))

j_thr <- youden_threshold(inner_cv$obs, inner_cv$HighSIB)

outer_j_thresholds[i] <- j_thr

probs <- predict(mod_inner, newdata = dtest, type = "prob")[, "HighSIB"]

preds_05 <- ifelse(probs >= 0.5, "HighSIB", "LowSIB")

preds_J <- ifelse(probs >= j_thr, "HighSIB", "LowSIB")

outer_preds[[i]] <- data.frame(

fold = i,

obs = dtest$Suicide,

prob = probs,

pred_05 = factor(preds_05, levels = c("LowSIB","HighSIB")),

pred_J = factor(preds_J, levels = c("LowSIB","HighSIB"))

)

}

outer_df <- dplyr::bind_rows(outer_preds)

outer_df$obs <- factor(outer_df$obs, levels = c("LowSIB","HighSIB"))

outer_df$pred_05 <- factor(outer_df$pred_05, levels = c("LowSIB","HighSIB"))

outer_df$pred_J <- factor(outer_df$pred_J, levels = c("LowSIB","HighSIB"))

cm_05 <- caret::confusionMatrix(outer_df$pred_05, outer_df$obs, positive = "HighSIB")

cm_J <- caret::confusionMatrix(outer_df$pred_J, outer_df$obs, positive = "HighSIB")

# ROC AUC with 95% CI (DeLong) across pooled outer test predictions

roc_obj <- pROC::roc(response = outer_df$obs, predictor = outer_df$prob,

levels = c("LowSIB","HighSIB"), direction = "<")

auc_outer <- as.numeric(pROC::auc(roc_obj))

ci_outer <- as.numeric(pROC::ci.auc(roc_obj)) # 95% CI

# AUPRC (area under Precision-Recall curve) – positive class = HighSIB

labels01 <- ifelse(outer_df$obs == "HighSIB", 1, 0)

pr_obj <- PRROC::pr.curve(scores.class0 = outer_df$prob[labels01 == 1],

scores.class1 = outer_df$prob[labels01 == 0],

curve = TRUE)

auprc_outer <- pr_obj$auc.integral

# Aggregate confusion matrices:

cm_05 <- caret::confusionMatrix(data = outer_df$pred_05, reference = outer_df$obs, positive = "HighSIB")

cm_J <- caret::confusionMatrix(data = outer_df$pred_J, reference = outer_df$obs, positive = "HighSIB")

# Pull common metrics

extract_metrics <- function(cm) {

data.frame(

Accuracy = cm$overall["Accuracy"],

Sens = cm$byClass["Sensitivity"],

Spec = cm$byClass["Specificity"],

PPV = cm$byClass["Pos Pred Value"],

NPV = cm$byClass["Neg Pred Value"]

)

}

metrics_05 <- extract_metrics(cm_05)

metrics_J <- extract_metrics(cm_J)

# Calibration: slope & intercept

logit_p <- qlogis(pmin(pmax(outer_df$prob, 1e-6), 1 - 1e-6))

cal_df <- data.frame(y = as.numeric(outer_df$obs == "HighSIB"), logit_p = logit_p)

cal_fit <- glm(y ~ logit_p, data = cal_df, family = binomial())

cal_intercept <- coef(cal_fit)[1]

cal_slope <- coef(cal_fit)[2]

cat("\n--- Nested CV (outer test) performance ---\n")

cat(sprintf("ROC AUC = %.3f (95%% CI %.3f–%.3f)\n", auc_outer, ci_outer[1], ci_outer[3]))

cat(sprintf("AUPRC = %.3f\n", auprc_outer))

cat("\nThreshold = 0.5\n"); print(metrics_05)

cat("\nThreshold = Youden J (per-fold, chosen on inner CV)\n"); print(metrics_J)

cat(sprintf("\nCalibration intercept = %.3f, slope = %.3f\n", cal_intercept, cal_slope))

###################################################################################

###################################################################################

###################################################################################

imaging_candidates <- c(

"LSMedFG","RrACC","RMidCing","RSTG","LSFG",

"RMFG9","RSFG10","RSFG9","LMedFG9","ACCThickness"

)

imaging_cols <- intersect(imaging_candidates, names(dat_final))

message("Imaging columns detected: ", paste(imaging_cols, collapse = ", "))

# Model 1 = clinical-only; Model 2 = clinical+imaging

dat30_m1 <- dat_final %>% dplyr::select(-dplyr::all_of(imaging_cols))

dat30_m2 <- dat_final

dat30_m1$Suicide <- factor(dat30_m1$Suicide, levels = c("LowSIB","HighSIB"))

dat30_m2$Suicide <- factor(dat30_m2$Suicide, levels = c("LowSIB","HighSIB"))

# Common outer folds (stratified)

set.seed(4242)

K_OUTER <- 5

outer_folds_idx <- createFolds(dat30_m2$Suicide, k = K_OUTER, returnTrain = FALSE)

# Inner-CV control

inner_ctrl <- trainControl(

method = "repeatedcv",

number = 5, repeats = 3,

classProbs = TRUE,

summaryFunction = twoClassSummary,

savePredictions = "final"

)

lambda_grid <- expand.grid(.alpha = 1, .lambda = seq(0.001, 0.1, by = 0.001))

youden_threshold <- function(truth, prob_pos) {

roc_obj <- pROC::roc(response = truth, predictor = prob_pos,

levels = c("LowSIB","HighSIB"), direction = "<")

as.numeric(pROC::coords(roc_obj, "best", ret = "threshold", best.method = "youden"))

}

run_nested <- function(data, folds_idx) {

y <- data$Suicide

outs <- vector("list", length(folds_idx))

for (i in seq_along(folds_idx)) {

test_idx <- folds_idx[[i]]

train_idx <- setdiff(seq_len(nrow(data)), test_idx)

dtrain <- data[train_idx, , drop = FALSE]

dtest <- data[test_idx, , drop = FALSE]

set.seed(100 + i)

mod_inner <- caret::train(

Suicide ~ ., data = dtrain, method = "glmnet",

trControl = inner_ctrl, tuneGrid = lambda_grid,

family = "binomial", metric = "ROC",

preProcess = c("center", "scale")

)

best_lambda <- mod_inner$bestTune$lambda

inner_cv <- mod_inner$pred %>%

dplyr::filter(lambda == best_lambda) %>%

dplyr::mutate(obs = factor(obs, levels = c("LowSIB","HighSIB")))

j_thr <- youden_threshold(inner_cv$obs, inner_cv$HighSIB)

probs <- predict(mod_inner, newdata = dtest, type = "prob")[, "HighSIB"]

preds_05 <- ifelse(probs >= 0.5, "HighSIB", "LowSIB")

preds_J <- ifelse(probs >= j_thr, "HighSIB", "LowSIB")

outs[[i]] <- data.frame(

row_id = test_idx, # keep original row index for pairing

fold = i,

obs = dtest$Suicide,

prob = probs,

pred_05 = factor(preds_05, levels = c("LowSIB","HighSIB")),

pred_J = factor(preds_J, levels = c("LowSIB","HighSIB")),

stringsAsFactors = FALSE

)

}

dplyr::bind_rows(outs)

}

# 2) Run both models on same folds #

outer_m1 <- run_nested(dat30_m1, outer_folds_idx) # clinical-only

outer_m2 <- run_nested(dat30_m2, outer_folds_idx) # clinical+imaging

# Align rows for paired tests

pair_df <- inner_join(

outer_m1 %>% dplyr::select(row_id, obs, prob_m1 = prob),

outer_m2 %>% dplyr::select(row_id, prob_m2 = prob),

by = "row_id"

)

pair_df$obs <- factor(pair_df$obs, levels = c("LowSIB","HighSIB"))

# 3) AUCs & paired ΔAUC #

roc_m1 <- pROC::roc(pair_df$obs, pair_df$prob_m1, levels = c("LowSIB","HighSIB"), direction = "<")

roc_m2 <- pROC::roc(pair_df$obs, pair_df$prob_m2, levels = c("LowSIB","HighSIB"), direction = "<")

auc_m1 <- as.numeric(pROC::auc(roc_m1))

auc_m2 <- as.numeric(pROC::auc(roc_m2))

delta_auc <- auc_m2 - auc_m1

# Bootstrap CI for ΔAUC (paired)

set.seed(2025)

B <- 2000

idx <- seq_len(nrow(pair_df))

boot_deltas <- replicate(B, {

s <- sample(idx, replace = TRUE)

roc1 <- pROC::roc(pair_df$obs[s], pair_df$prob_m1[s], levels = c("LowSIB","HighSIB"), direction = "<")

roc2 <- pROC::roc(pair_df$obs[s], pair_df$prob_m2[s], levels = c("LowSIB","HighSIB"), direction = "<")

as.numeric(pROC::auc(roc2) - pROC::auc(roc1))

})

ci_delta <- quantile(boot_deltas, probs = c(0.025, 0.975), na.rm = TRUE)

# 4) Confusion matrices (0.50 & Youden J) #

# Aggregate across all outer test predictions for each model

agg_cm <- function(outer_df, threshold_col) {

df <- outer_df %>%

mutate(obs = factor(obs, levels = c("LowSIB","HighSIB")))

cm <- caret::confusionMatrix(df[[threshold_col]], df$obs, positive = "HighSIB")

counts <- table(Predicted = df[[threshold_col]], Reference = df$obs)

list(cm = cm, counts = counts)

}

cm_m1_05 <- agg_cm(outer_m1, "pred_05")

cm_m1_J <- agg_cm(outer_m1, "pred_J")

cm_m2_05 <- agg_cm(outer_m2, "pred_05")

cm_m2_J <- agg_cm(outer_m2, "pred_J")

# 5) Print a compact summary #

cat("\n=== Paired comparison on n = 30 (same folds) ===\n")

cat(sprintf("Model 1 AUC = %.3f, Model 2 AUC = %.3f, ΔAUC = %.3f\n", auc_m1, auc_m2, delta_auc))

cat(sprintf("Bootstrap 95%% CI for ΔAUC: [%.3f, %.3f]\n", ci_delta[1], ci_delta[2]))

#######################################################################

#######################################################################

#######################################################################

grp_raw <- dat0$Group

grp_chr <- if (inherits(grp_raw, "labelled")) haven::as_factor(grp_raw) else grp_raw

grp_chr <- tolower(trimws(as.character(grp_chr)))

is_high <- grepl("\\bhigh\\b", grp_chr) |

grp_chr %in% c("1","yes","y","hi","high sib","high_sib","highsib")

is_low <- grepl("\\blow\\b", grp_chr) |

grp_chr %in% c("0","no","n","lo","low sib","low_sib","lowsib")

Suicide_clean <- ifelse(is_high, "HighSIB",

ifelse(is_low, "LowSIB", NA_character_))

Suicide_clean <- factor(Suicide_clean, levels = c("LowSIB","HighSIB"))

dat_base <- dat_final

dat_base$Suicide <- Suicide_clean

# Sanity check: both classes must be present

tbl_out <- table(dat_base$Suicide, useNA = "ifany"); print(tbl_out)

stopifnot(!any(is.na(dat_base$Suicide)), all(tbl_out > 0))

# Repeated 5x3 CV with out-of-fold predictions saved

set.seed(2025)

ctrl_rep <- caret::trainControl(

method = "repeatedcv",

number = 5, repeats = 3,

classProbs = TRUE,

summaryFunction = twoClassSummary,

savePredictions = "final"

)

# NegUrg only

m_baseline_nu <- caret::train(

reformulate(termlabels = "NegUrgency", response = "Suicide"),

data = dat_base, method = "glm", family = binomial(),

trControl = ctrl_rep, metric = "ROC"

)

# Hostility only

m_baseline_host <- caret::train(

reformulate(termlabels = "P7Hostility", response = "Suicide"),

data = dat_base, method = "glm", family = binomial(),

trControl = ctrl_rep, metric = "ROC"

)

# Pooled AUCs from saved predictions + DeLong 95% CIs

get_auc_ci <- function(mod) {

df <- as.data.frame(mod$pred)

df$obs <- factor(df$obs, levels = c("LowSIB","HighSIB"))

# caret names the positive-class probability column after the level itself

pos_col <- if ("HighSIB" %in% names(df)) {

"HighSIB"

} else {

meta <- c("obs","pred","rowIndex","Resample")

cand <- setdiff(names(df), meta)

num_cand <- cand[sapply(df[cand], function(x) is.numeric(x) || is.double(x))]

if (length(num_cand) > 0) tail(num_cand, 1) else tail(cand, 1)

}

roc_obj <- pROC::roc(response = df$obs,

predictor = df[[pos_col]],

levels = c("LowSIB","HighSIB"),

direction = "<")

c(

AUC = as.numeric(pROC::auc(roc_obj)),

CI_low = as.numeric(pROC::ci.auc(roc_obj))[1],

CI_high = as.numeric(pROC::ci.auc(roc_obj))[3]

)

}

nu_auc <- get_auc_ci(m_baseline_nu)

host_auc <- get_auc_ci(m_baseline_host)

baseline_tbl <- tibble(

Model = c("NegUrg only", "Hostility only"),

AUC = c(nu_auc["AUC"], host_auc["AUC"]),

CI = sprintf("[%.3f, %.3f]",

c(nu_auc["CI_low"], host_auc["CI_low"]),

c(nu_auc["CI_high"], host_auc["CI_high"]))

)

print(baseline_tbl)
